# Association Between Continuous Consumption of Yogurt Fermented with *Lactobacillus delbrueckii* ssp. *bulgaricus* OLL1073R-1, Salivary Antimicrobial Proteins, and Tongue-Coating Microbiota: An Observational Human Study

**DOI:** 10.3390/jcm15031244

**Published:** 2026-02-04

**Authors:** Yuko Yamamoto, Toshiya Morozumi, Takehiro Yokoo, Toru Takahashi, Juri Saruta, Hanae Tsuchihashi, Hiroki Negishi, Junko Mochizuki, Yuta Hosomi, Wakako Sakaguchi, Masahiro To, Seiya Makino, Hiroshi Kano, Kenichi Hojo, Keiichi Tsukinoki

**Affiliations:** 1 Department of Dental Hygiene, Kanagawa Dental University, Junior College, Yokosuka 238-8580, Japan; 2 Department of Endodontics, The Nippon Dental University School of Life Dentistry at Niigata, Niigata 951-8580, Japan; morozumi@ngt.ndu.ac.jp; 3 Health Science Research Unit, R&D Division, Meiji Co., Ltd., Tokyo 192-0919, Japan; takehiro.yokoo@meiji.com (T.Y.); junko.mochizuki@meiji.com (J.M.); seiya.makino@meiji.com (S.M.); kenichi.houjou@meiji.com (K.H.); 4 Faculty of Pharmaceutical and Medical Sciences, Nihon Pharmaceutical University, Ina 362-0806, Japan; t-takahashi@nichiyaku.ac.jp; 5 Department of Education Planning, Kanagawa Dental University, Yokosuka 238-0003, Japan; saruta@kdu.ac.jp; 6 Lactic Acid Bacteria & Fermentation Technology Research Unit, R&D Division, Meiji Co., Ltd., Tokyo 192-0919, Japan; hanae.tsuchihashi@meiji.com; 7 Wellness Science Labs, Meiji Holdings Co., Ltd., Tokyo 192-0919, Japan; hiroki.negishi.aa@meiji.com (H.N.); hiroshi.kano@meiji.com (H.K.); 8 Department of Environmental Pathology, Kanagawa Dental University, Yokosuka 238-0003, Japan; hosomi@kdu.ac.jp (Y.H.); sakaguchi@kdu.ac.jp (W.S.); 9 Department of Nursing, School of Health Sciences, Tokyo University of Technology, Tokyo 144-8535, Japan; tohmshr@stf.teu.ac.jp

**Keywords:** saliva, yogurt fermented with *Lactobacillus delbrueckii* ssp. *bulgaricus* OLL1073R-1, salivary antimicrobial proteins, tongue-coating microbiota, upper respiratory tract infection

## Abstract

**Background/Objectives**: Oral microbial homeostasis is crucial for overall health. Nonetheless, the relationship between probiotics and the oral environment remains unclear. This study investigated the association between continuous consumption of yogurt containing *Lactobacillus delbrueckii* ssp. *bulgaricus* OLL1073R-1 (LbR1 yogurt), salivary antimicrobial proteins (AMPs), tongue-coating microbiota, and upper respiratory tract infection (URTI) frequency. **Methods**: This observational study was conducted on 53 nursing home care workers, categorized into a group who consumed LbR1 yogurt daily for over 1 year (*n* = 40, yogurt group) and a non-intake group (*n* = 13, non-yogurt group). Salivary and tongue-coating samples were collected. **Results**: The yearly URTI frequency was lower in the yogurt group than in the non-yogurt group (*p* = 0.003). The salivary β-defensin-2 (HBD2) and β-defensin-3 (HBD3) flow rates were higher in the yogurt group than in the non-yogurt group (*p* = 0.02 and *p* = 0.0009, respectively). *Fusobacterium nucleatum* (*F. nucleatum*) ssp. *animalis* abundance was lower in the yogurt group than in the non-yogurt group (*p* = 0.04). Bayesian network analysis indicated an association between yogurt consumption and the yearly URTI frequency and salivary HBD2 and HBD3 flow rates. **Conclusions**: Continuous consumption of LbR1 yogurt was associated with elevated salivary HBD2 and HBD3, reduced abundance of *F. nucleatum* ssp. *animalis*, and decreased URTI frequency. Thus, LbR1 yogurt intake is associated with modulated oral immunity and microbiota, suggesting a potential link to reduced URTIs. However, as an observational pilot study, its results should be interpreted with caution.

## 1. Introduction

As a gateway to the digestive system, the oral cavity harbors the second most diverse microbial community in the human body, comprising over 700 bacterial species forming a complex ecosystem [1]. The oral microbiota is a crucial intermediary between the host and the external environment, contributing to essential physiological systems while acting as a defensive barrier against pathogens [2]. Recently, evidence demonstrates that the oral microbiome significantly impacts overall health, uncovering a bidirectional communication network known as the oral–gut axis [3]. Oral microbiota dysbiosis is associated with various systemic diseases, including those affecting the respiratory, gastrointestinal, and cardiovascular systems, as well as certain cancers [4], thereby suggesting that maintaining the homeostasis of oral microorganisms is important for both local and overall health [5].

Probiotics are live microorganisms that confer health benefits to the host when administered in adequate amounts; moreover, they are effective for regulating the microbial ecosystem on the intestinal mucosal surface and maintaining health [6]. The action mechanisms of probiotics are multifaceted, including pathogen competitive exclusion, antimicrobial substance production, and cytokine induction [7]. Lactic acid bacteria are the most extensively studied group of probiotics and are widely used in fermented foods such as yogurt [8]. Recent studies suggest that probiotics may improve oral health by reducing pathogenic bacteria and help prevent systemic infections, such as upper respiratory tract infections (URTIs), by regulating the host immune system via the gut-lung axis [6]. Despite these promising findings, the precise mechanisms linking oral probiotic intake to oral environment changes and subsequent systemic health remain unclear [9].

The tongue coating is a primary reservoir for oral microorganisms, releasing them into the saliva [10]. Its composition is influenced and altered by dietary interventions [11], and tongue-coating microbiota has become a digestive health indicator [12]. Furthermore, saliva, the oral cavity secretory fluid, contains a series of antimicrobial proteins (AMPs), including human β-defensin (HBD) and lactoferrin, which form the first line of the natural defense of the oral mucosa [13]. Some clinical trials have examined the effects of probiotics on oral bacteria associated with dental caries and periodontal disease; however, their impact on tongue-coating microbiota composition remains largely unexplored [9]. Nevertheless, probiotic intake-mediated changes in the bacterial flora of the tongue coating and their interaction with AMP activity in the saliva represent an important yet understudied area [9]. In the super-aged society of Japan, studying these interactions within the oral cavity is essential for developing nutritional strategies to enhance host defenses.

In our previous study, we demonstrated that continuous consumption of yogurt fermented with *Lactobacillus delbrueckii* ssp. *bulgaricus* OLL1073R-1 (LbR1 yogurt) by elderly individuals resulted in elevated levels of salivary IgA flow rate and influenza virus-bound salivary IgA [14,15]. Furthermore, LbR1 yogurt intake activates natural killer (NK) cells [16]. Therefore, this study aimed to investigate the effects of continuous consumption of LbR1 yogurt on the oral and systemic health of adult volunteers. We hypothesized that the regular intake of LbR1 yogurt would modulate the oral environment and host immune responses. Specifically, we analyzed the effect on the salivary AMP flow rates, including HBD2, HBD3, lactoferrin, immunoglobulin A (IgA), and lysozyme, as well as tongue-coating microbiota composition, salivary flow rate, and URTI frequency.

## 2. Materials and Methods

### 2.1. General Data

This observational study was conducted among nursing home care workers. The participants were non-smoking nursing care workers aged 20 years or older who had been working at the nursing home “Samukawa Home” (Kanagawa, Japan) for more than 1 year as of October 2023. For approximately 10 years, this nursing home has provided 112 g of LbR1 yogurt (Meiji probio yogurt R-1; Meiji Co., Ltd., Tokyo, Japan) per day to nursing care workers who request it. Yogurt is defined as a clotted milk product that results from the lactic acid fermentation in milk by *Lactobacillus delbrueckii* ssp. *bulgaricus* and *Streptococcus thermophilus* [17]. This yogurt is manufactured using the LbR1 strain and *Streptococcus thermophilus* OLS3059. Nursing care workers consume LbR1 yogurt upon arriving at the nursing home, and on their days off, they are provided with LbR1 yogurt to take home. The intake of LbR1 yogurt is supervised by the nursing home director. Participants either consumed LbR1 yogurt daily for more than 1 year (yogurt group) or did not consume LbR1 yogurt (non-yogurt group). Overall, 53 participants (40 and 13 in the yogurt and non-yogurt groups, respectively) were recruited and included in the analysis. The sex, age, body mass index, daily sleep duration, number of coronavirus disease 2019 (COVID-19) infections (total), number of URTI (number of times a doctor diagnosed URTI at the hospital, within the past year), and current subjective health status of the participants (evaluated in increments of 0.1, on a scale ranging from −7 [most unhealthy] to +7 [most healthy]) were assessed via a questionnaire. The nursing home director verified the number of URTI episodes reported by participants in the questionnaire. A dental hygienist recorded the number of teeth in the mouth and confirmed through interviews that all participants brushed their teeth at least twice a day.

### 2.2. Ethical Approval

This study was conducted in accordance with the Declaration of Helsinki and approved by the Research Ethics Committee of Kanagawa Dental University (3 July 2023, Reference Number 942). All participants received written and verbal explanations regarding the research content, assurance of anonymity, use of data, and risks associated with participation, and they provided written informed consent.

### 2.3. Collecting Saliva

Saliva was collected from participants using the same method as in our previous study and frozen until analysis [18]. Participants were requested to avoid strenuous exercise and alcohol consumption the day before. Participants completed all eating, drinking, and toothbrushing one hour prior to saliva collection and remained seated and at rest for 15 min before sampling. Saliva samples were collected using the Salivette^®^ (SARSTEDT AG & Co. KG, Nümbrecht, Germany) between 9:00 and 10:00 a.m. A sponge made of polypropylene–polyethylene polymer was held under the tongue for 2 min, and the saliva-containing sponge was returned to the tube. The tubes were quickly ice-cooled, centrifuged (1500× *g*, 20 min, 4 °C), and stored at −80 °C until analysis.

### 2.4. Collecting Tongue Coating

Tongue coatings were collected by the participants themselves, under the supervision of dental hygienists, using sterile cotton swabs. The individuals watched a video on how to collect tongue coating and practiced it beforehand. The tongue coating was collected by placing a sterile cotton swab at the center of the right side of the tongue, applying pressure until the handle bent slightly, and moving the swab back and forth three times across the tongue width. Following tongue coating collection, the cotton swab was placed in a plastic tube and stored at −80 °C until analysis.

### 2.5. Salivary Protein Quantitation

The concentrations of salivary AMPs were measured using enzyme-linked immunosorbent assay (ELISA) kits. Total IgA concentration was analyzed using a Human IgA ELISA kit (Fortis Life Sciences, LLC., Boston, MA, USA). HBD2 concentration was assessed using Human β Defensin-2 ELISA Kit (Arigo Biolaboratories Corp., Zhubei City, Hsinchu County, Taiwan). HBD3 concentration was analyzed using the Human DEFB-3/Beta-defensin 103/BD-3 ELISA Kit (RayBiotech, Inc., Peachtree Corners, GA, USA). Lactoferrin concentration was assessed using the AssayMax Human Lactoferrin ELISA Kit (AssayPro LLC., St. Charles, MO, USA). The lysozyme concentration was measured using the AssayMax Human Lysozyme ELISA Kit (AssayPro LLC., St. Charles, MO, USA). The analyses were performed according to the instructions provided for each ELISA kit. The flow rate per minute (min) for each antimicrobial protein was calculated by multiplying the concentration by the salivary flow rate.

### 2.6. DNA Extraction from Tongue-Coating Samples

DNA was extracted from tongue-coating samples according to Protocol Q reported by Costea et al. [19], with slight modifications using the QIAGEN QIAamp Fast DNA Stool Mini Kit (QIAGEN N.V., Venlo, Limburg, The Netherlands). Specifically, the tip of the swab was aseptically cut and placed into a screw-cap tube containing 0.3 g of zirconia beads with a diameter of 0.1 mm and 1 mL of QIAGEN Buffer ASL (QIAGEN). The samples were incubated at 95 °C for 15 min. Subsequently, bacterial cell lysis was carried out using FastPrep-24 5G (MP Biomedicals LLC., Irvine, CA, USA) for 4 min, employing a cycle of 1 min beating and 5 min resting, repeated four times.

### 2.7. Analysis of Tongue-Coating Microbiota

Library preparation using DNA extracted from tongue-coating samples was performed according to the Illumina 16S Metagenomic Sequencing Library Preparation protocol (Illumina Inc., San Diego, CA, USA). Because oral bacteria, especially subspecies of *Fusobacterium*, are better analyzed using the V1–V2 region among the 16S rRNA variable regions, we selected the V1–V2 region for amplification [20]. The V1–V2 regions were amplified using primers as described in Allali et al. [21] (Forward: TCGTCGGCAGCGTCAGATGTGTATAAGAGACAGAGAGTTTGATCCTGGCTCAG, Reverse: GTCTCGTGGGCTCGGAGATGTGTATAAGAGACAGGCTGCCTCCCGTAGGAGT). The library was sequenced using a MiSeq v2 kit (500 cycles). Sequence data were analyzed with the QIIME 2 pipeline (version 2020.5) [22]. Raw reads were quality-filtered, denoised, and chimeric sequences were removed using DADA2 implemented in QIIME2. After quality filtering and merging, the mean sequence length was 310 bp. After quality control, samples contained a minimum of 33,888 reads. All samples were rarefied to 10,000 reads per sample for downstream diversity analyses to account for differences in sequencing depth. Taxonomic identification was conducted using a classifier trained with the eHOMD 16S rRNA RefSeq Version 15.22 database [23].

### 2.8. Bayesian Network Analysis

A Bayesian network is a probabilistic graphical model that represents variables and their conditional dependencies using a directed acyclic graph [24]. In clinical and epidemiological research, Bayesian network analysis serves as an established framework for causal inference [25]. Furthermore, Bayesian networks are categorized as machine learning rather than statistical hypothesis testing [26]. Therefore, Bayesian network analysis is robust to confounding [27]. In the present study, Bayesian network analysis was conducted as described in our previous study [28].

### 2.9. Statistical Analysis

All statistical analyses were performed using JMP (version 12; JMP Statistical Discovery LLC., Cary, NC, USA) and R (version 4.3.2; The R Project for Statistical Computing, Vienna, Austria). The Mann–Whitney U test was used to compare the two groups. Since this study is an observational study, the statistical analysis of the tongue-coating microbiota was conducted in an exploratory manner. Adopting unadjusted *p*-values without FDR correction is considered acceptable in the exploratory phase of a study [29]. Therefore, in the present study, unadjusted *p*-values were adopted without False Discovery Rate (FDR) correction for the Mann–Whitney U tests regarding the tongue-coating microbiota. Correlation analysis was performed using Spearman’s rank correlation coefficient. Tongue-coating microbiota was evaluated using α diversity and β diversity. α diversity, representing within-individual microbial diversity, and β diversity, indicating between-individual microbial similarity, were analyzed using QIIME2. α diversity was assessed with the Chao1 and Shannon indices, whereas β diversity was measured by performing principal coordinate analysis (PCoA) on unweighted and weighted UniFrac distance matrices in R (vegan package). Differences in β diversity between the groups were identified using permutational multivariate analysis of variance (PERMANOVA), implemented in R (adonis2 function, vegan package). α and β diversity visualization was performed using GraphPad Prism (version 10.1.2; GraphPad Software, Boston, MA, USA) and R (ggplot2 package). Causal relationships between the variables were analyzed using Bayesian network analysis. The level of statistical significance was set at *p* < 0.05.

## 3. Results

### 3.1. General Data of Participants

The general data of the participants are presented in Table 1. The yogurt group was older than the non-yogurt group (*p* = 0.03). The yearly URTI frequency was lower in the yogurt group than in the non-yogurt group (*p* = 0.003). No difference was observed in the salivary flow rate between the two groups (*p* = 0.6). Similarly, no differences were observed between the two groups in any of the other parameters.

### 3.2. Tongue-Coating Microbiota Diversity

**Figure 1:**
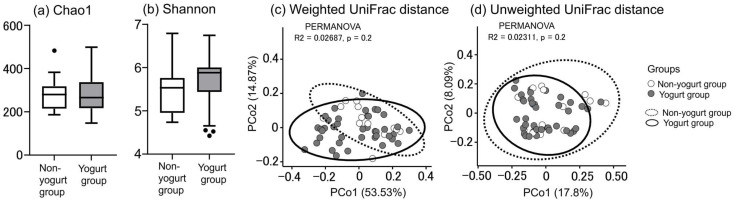
α diversity Chao1 index (**a**), α diversity Shannon index (**b**), β diversity weighted UniFrac distance (**c**), and β diversity unweighted UniFrac distance (**d**). *n* = 40, yogurt group; *n* = 13, non-yogurt group. (**a**,**b**): Box plots display median, first quartile, third quartile, minimum, and maximum values. Significant differences in α diversity were determined using the Mann–Whitney U test. There were no differences between the two groups in the Chao1 and Shannon indices (*p* = 1.0 and 0.09, respectively). (**c**,**d**): Significant differences in β diversity were determined using permutational multivariate analysis of variance (PERMANOVA). There were no differences between the two groups in the weighted and unweighted UniFrac distances (*p* = 0.2 and 0.2, respectively). PERMANOVA, Permutational Multivariate Analysis of Variance.

The α diversity of the tongue-coating microbiota did not differ significantly between the two groups, as measured by both the Chao 1 index and the Shannon index (*p* = 1.0, 0.09, respectively, Figure 1). PCoA based on unweighted and weighted UniFrac distance metrics demonstrated no significant differences in tongue-coating microbiota composition between the two groups (*p* = 0.2 for both metrics, Figure 1).

### 3.3. Tongue-Coating Microbiota Composition

**Figure 2:**
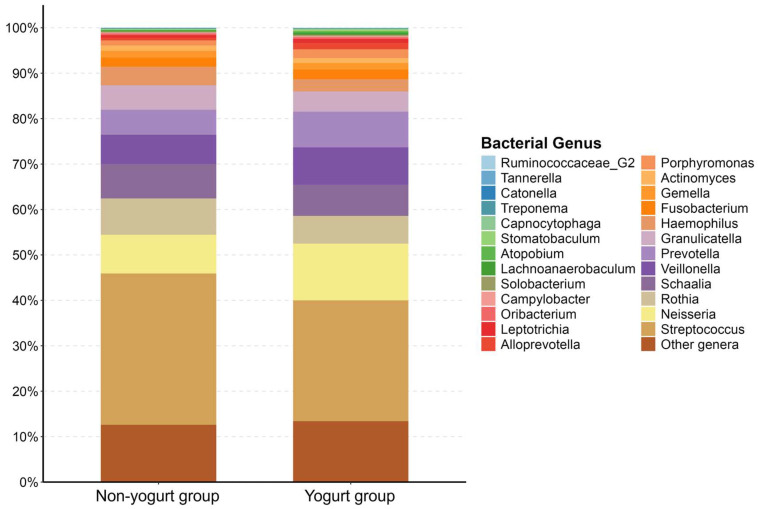
Composition of the tongue-coating microbiota of the yogurt and non-yogurt groups. The results are displayed as 100% stacked bar charts. Bacterial genus composition in tongue-coating samples from the non-yogurt and yogurt groups is indicated in panels.

The tongue coating microbiota of the participants contained 57 bacterial genera present at a proportion of 0.01% or higher. The proportions of the top 25 bacterial genera are displayed in Figure 2. Regardless of the yogurt consumption, the most abundant bacteria at the genus level were *Streptococcus*, *Neisseria*, *Veillonella*, *Prevotella*, *Rothia*, and *Schaalia*.

Table 2 presents the top 25 genera of tongue-coating microbiota analyzed using 16S rRNA gene sequencing. The abundances of *Lachnoanaerobaculum* and Ruminococcaceae G2 were higher in the yogurt group than in the non-yogurt group (*p* = 0.004, 0.004, respectively). There were no differences in the relative abundance of other bacteria between the two groups (Table 2).

### 3.4. Fusobacterium Species Abundances in the Tongue-Coating Microbiota

The abundances of the major species of *Fusobacterium* in tongue coating are shown in Table 3. *Fusobacterium nucleatum* ssp. *animalis* abundance was lower in the yogurt group than in the non-yogurt group (*p* = 0.04). No differences were observed between the two groups for other species (Table 3).

### 3.5. Salivary AMPs Flow Rate

**Figure 3:**
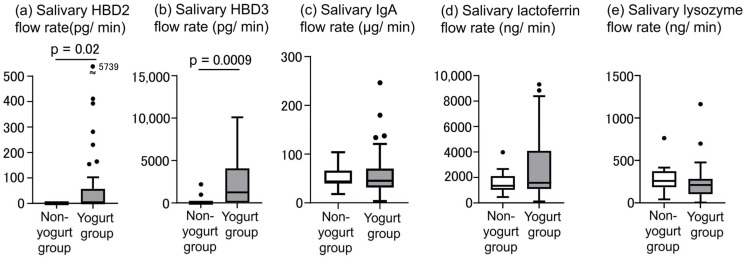
Flow rates of salivary β-defensin-2 (HBD2) (**a**), salivary β-defensin-3 (HBD3) (**b**), salivary IgA (**c**), salivary lactoferrin (**d**), and salivary lysozyme (**e**). *n* = 40, yogurt group; *n* = 13, non-yogurt group. Box plots display median, first quartile, third quartile, minimum, and maximum values. Significant differences were determined using the Mann–Whitney U test. Significant differences were observed between the two groups in the salivary HBD2 and HBD3 flow rates (*p* = 0.02 and 0.0009, respectively). There were no differences between the two groups in the salivary IgA, lactoferrin, and lysozyme flow rates (*p* = 0.8, 0.5, and 0.2, respectively).

The salivary HBD2 and HBD3 flow rates were higher in the yogurt group than in the non-yogurt group (*p* = 0.02, 0.0009, respectively, Figure 3). There were no differences in the flow rates of salivary IgA, lactoferrin, and lysozyme between the two groups (*p* = 0.8, 0.5, and 0.2, respectively, Figure 3).

### 3.6. Causal Analysis Using a Bayesian Network

**Figure 4:**
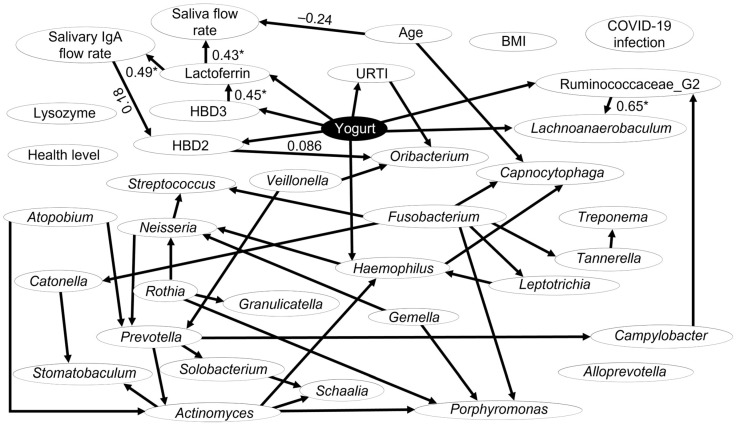
Bayesian network depicting the causal relationships among the factors. A total of 36 items were analyzed: six general participant data (Table 1), five salivary antimicrobial proteins, and 25 genera dominating the tongue-coating microbiota. Yogurt: consumption of yogurt fermented with *Lactobacillus delbrueckii* ssp. *bulgaricus* OLL1073R-1; Lactoferrin: salivary lactoferrin flow rate; HBD2: salivary β-defensin-2 flow rate; HBD3: salivary β-defensin-3 flow rate; Lysozyme: salivary lysozyme flow rate; BMI: body mass index; URTI: upper respiratory tract infection; COVID-19 infection: total number of Coronavirus Disease 2019 infections; Health level: current subjective health status. Arrows point from cause (source) to effect (destination). Numbers alongside arrows indicate Spearman’s rank correlation coefficients (*n* = 53). Statistical superiority, defined as *p* < 0.05, is denoted with *.

Focusing on the general data of the participants (Table 1), salivary AMPs (Figure 3), and the top 25 bacterial genera in the tongue-coating microbiota (Table 2), we analyzed the causal relationships among these factors using a Bayesian network (Figure 4). To confirm the impact of the higher median age in the yogurt group compared to that in the non-yogurt group on other factors, we also implemented age as a factor and performed the analysis.

The Bayesian network analysis revealed that LbR1 yogurt consumption affected the yearly URTI frequency. Additionally, it modulated salivary AMP flow rates, specifically those of HBD2, HBD3, and lactoferrin. The salivary AMPs demonstrated sequential effects: the HBD3 flow rate affected lactoferrin flow rate, lactoferrin flow rate influenced IgA flow rate, and IgA flow rate modulated HBD2 flow rate. LbR1 yogurt intake affected the salivary flow rate via the lactoferrin flow rate. Age influenced the salivary flow rate.

The analysis revealed that LbR1 yogurt intake affected *Lachnoanaerobaculum*, Ruminococcaceae G2, and *Haemophilus* in the tongue coating, as well as the *Oribacterium* in the tongue coating via the salivary HBD2 flow rate. Furthermore, age directly influenced two factors: saliva flow rate and the proportion of *Capnocytophaga* in tongue coating and did not exert any direct or indirect influence on the other factors.

## 4. Discussion

Recently, the oral microbiota has gained attention in the context of the “oral–gut axis,” which highlights the close relationship between oral and gut health [30]. Probiotics can restore the gut microbial composition and exert beneficial effects on the intestines [31]. In addition, probiotics have also been suggested to contribute not only to gut health but also to oral health and to the prevention of infections such as URTIs [6]. However, the underlying mechanisms remain unclear. Lactic acid bacteria are the primary probiotic organisms [32], and yogurt is a representative fermented food produced by lactic acid bacteria. To the best of our knowledge, this study is the first to analyze the relationship among yogurt consumption, salivary AMPs, tongue-coating microbiota, and URTIs.

In this study, a significant age disparity was observed between the groups; the yogurt group was approximately 15 years older than the non-yogurt group (Table 1). To address this confounding variable, we incorporated age as a factor in our Bayesian network analysis. The results revealed that age directly influenced only the salivary flow rate and the proportion of *Capnocytophaga* in the tongue-coating microbiota, and did not exert any direct or indirect influence on other factors (Figure 4). However, as this study is observational, the results should be interpreted with caution.

In the present study, Bayesian network analysis revealed that LbR1 yogurt consumption may have influenced salivary AMP secretion rates, specifically those of HBD2, HBD3, and lactoferrin, with HBD2 and HBD3 exhibiting elevated rates (Figure 3 and Figure 4). HBDs are peptides produced by epithelial cells that exert antimicrobial activity and can induce immune cell responses [33]. While they are typically present at low levels, their expression is upregulated in response to inflammatory cytokines, such as interleukin (IL)-1β and tumor necrosis factor (TNF)-α, resulting in enhanced antimicrobial activity [33]. Salivary HBD2 and HBD3 are produced by salivary duct cells, oral epithelial cells, and certain blood cells [34]. When *Limosilactobacillus reuteri* (formerly *Lactobacillus reuteri*) is administered to rats, bioactive molecules such as peptidoglycan in the cell wall of *L. reuteri* can activate the nucleotide-binding oligomerization domain-2 receptor within parotid gland epithelial cells, thereby increasing salivary HBD2 levels [35]. Furthermore, in experiments using human peripheral blood mononuclear cells, co-culturing six different lactic acid bacteria increased IL-1β and TNF-α production by all strains [36]. Notably, the increases in salivary HBD2 and HBD3 secretion rates observed in the yogurt group in our study may be attributed to the LbR1 cell wall acting on oral epithelial cells and to the increased production of IL-1β and TNF-α following LbR1 intake. As HBD2 and HBD3 exhibit distinct antimicrobial spectra and immune-regulatory functions [33], their biological relevance is noteworthy. Specifically, HBD2 exhibits strong activity primarily against Gram-negative bacteria, whereas HBD3 is effective against a broader range of microorganisms than HBD2, possessing broad activity against both Gram-positive bacteria and Gram-negative bacteria [33]. Moreover, HBD3 possesses antiviral properties against the influenza virus [37]. Consequently, their simultaneous high salivary secretion in response to LbR1 yogurt consumption may promote a robust innate immune response. These findings suggest that LbR1 yogurt consumption may contribute to maintaining oral immune defense not only in nursing home care workers but potentially also in populations with attenuated innate immunity. Moreover, Bayesian network analysis revealed that LbR1 yogurt intake influenced the salivary lactoferrin secretion rate. While no significant difference was observed between the groups, the median value was higher in the yogurt group than in the non-yogurt group (Figure 3 and Figure 4). Importantly, lactoferrin is an iron-binding glycoprotein found in human secretions, exerting antibacterial effects by depriving bacteria of iron required for proliferation and playing antiviral, anti-inflammatory, and immune-modulating roles [38]. Upon recognizing cell wall components of lactic acid bacteria (e.g., peptidoglycan) by pattern recognition receptors on neutrophils in the oral mucosa, neutrophil degranulation is induced, releasing granule-stored lactoferrin into saliva [39]. Therefore, the action of LbR1 cell wall components may have increased the salivary lactoferrin secretion rate in the yogurt group. Notably, our results indicated that LbR1 yogurt intake may have contributed to the elevated levels of salivary lactoferrin. Although natural killer (NK) cells activity was not measured in this study, a previous study by Kuhara et al. has demonstrated that oral administration of bovine lactoferrin to mice increases the number of NK cells in the peripheral blood and spleen [40]. Thus, it is biologically plausible that the elevated salivary lactoferrin concentration observed in the continuous LbR1 yogurt intake group reflects a similar modulation; however, based on our current data, this remains speculative. Additionally, Bayesian network analysis revealed that LbR1 yogurt intake did not affect the salivary secretion rates of IgA or lysozyme, with no significant differences observed between the two groups in either parameter (Figure 3 and Figure 4). IgA is the primary antibody responsible for the first line of defense on mucosal surfaces, including the digestive tract, airways, and oral cavity, where it neutralizes pathogens and prevents their entry into the body [41]. Previously, we found that consuming LbR1 yogurt increased the salivary IgA flow rate among nursing home residents [14]. Nevertheless, even in healthy individuals, salivary IgA levels decrease with age [42]; therefore, although LbR1 yogurt consumption may mitigate the age-related decline in salivary IgA levels, no difference was observed in this study between the two groups. Lysozyme is among the primary salivary antibacterial proteins, part of the innate immune system, breaking down bacterial cell walls [43]. Salivary lysozyme levels increase in response to systemic diseases and inflammatory states [43]. Therefore, salivary lysozyme flow rate may not have increased with LbR1 yogurt intake.

There was no difference in the proportion of major *Fusobacterium* species in the tongue coating between the yogurt and non-yogurt groups (Table 3). Nevertheless, the proportion of *F. nucleatum* ssp. *animalis* was lower in the yogurt group than in the non-yogurt group (Table 3). Notably, *F. nucleatum* is a Gram-negative anaerobic bacterium that is universally abundant in healthy oral microbiota [44]. Conversely, *F. nucleatum* ssp. *animalis* is particularly prevalent in colorectal cancer tumor tissues and is specialized in surviving in the highly acidic stomach environment [45]. Moreover, the continuous consumption of lactic acid bacteria-fermented dairy products (curds) increases salivary pH [46]. Therefore, *F. nucleatum* ssp. *animalis* failed to adapt to the oral environment, where pH increased due to LbR1 yogurt consumption, likely resulting in a reduced proportion of *F. nucleatum* ssp. *animalis* tongue coating in the yogurt group. Importantly, *F. nucleatum* ssp. *animalis* exhibits a strong tropism for inflammatory environments [47], whereas *L. reuteri* consumption reduces oral cavity inflammation [48]. Thus, LbR1 yogurt intake might potentially mitigate oral inflammation, possibly contributing to the decreased proportion of *F. nucleatum* ssp. *animalis* within the tongue-coating microbiota.

In the present study, Bayesian network analysis revealed that LbR1 yogurt consumption may have affected the abundance of *Lachnoanaerobaculum* and Ruminococcaceae G2 (belonging to the family Ruminococcaceae [49]) within the tongue-coating microbiota. Notably, both were more abundant in the yogurt group than in the non-yogurt group (Table 2 and Figure 4). *Lachnoanaerobaculum* and Ruminococcaceae are commensal bacteria found in the healthy oral cavities [50,51]; they are strictly anaerobic and are primary producers of butyric acid from acetic acid [50,52]. Furthermore, *Veillonella* levels in the tongue coating were higher in the yogurt group than in the non-yogurt group, although the difference was not significant (Table 3). Importantly, *Veillonella* produces acetic acid from lactic acid [53], which *Lachnoanaerobaculum* and Ruminococcaceae then use to produce butyric acid [50,51]. By providing the lactic acid from lactic acid bacteria, LbR1 yogurt consumption likely first increased *Veillonella* within the tongue coating, thereby converting lactic acid into acetic acid, and, concurrently, it may have promoted increases in *Lachnoanaerobaculum* and Ruminococcaceae, which metabolize acetic acid to butyric acid. While butyric acid produced by periodontal pathogens can reach extremely high concentrations in closed spaces such as periodontal pockets and promotes the pathogenesis of periodontal diseases [54], in this study, the proportions of *Lachnoanaerobaculum* and Ruminococcaceae G2 were very low (Table 2). Consequently, the butyrate produced would have been diluted in saliva [55], resulting in low concentrations that can suppress inflammation in macrophages, fibroblasts, and oral epithelial cells [56]. Therefore, LbR1 yogurt consumption may be associated with higher proportions of *Lachnoanaerobaculum* and Ruminococcaceae G2 in the tongue-coating microbiota, potentially enhancing butyrate production and contributing to inflammation suppression in the oral mucosa.

No difference in bacterial diversity within the tongue coating was observed between the yogurt and non-yogurt groups (Figure 1). Similarly, the tongue-coating bacterial abundance between the LbR1 yogurt group and the non-yogurt group demonstrated no significant differences, except for *Lachnoanaerobaculum* and Ruminococcaceae G2 (Table 3). The oral microbiota is a stable and mature ecosystem in which diverse bacteria coexist [57]. Genera with high prevalence, such as *Streptococcus*, *Neisseria*, and *Veillonella*, are highly adapted to and stable within the tongue coating environment, remaining resistant to minor external interventions [58]. Accordingly, LbR1 yogurt consumption stimulated the tongue-coating microbiota and altered the abundance of some low-prevalence bacteria but did not provide a sufficiently potent stimulus to modify the abundance of genera that were already highly stable.

Our results indicate that LbR1 yogurt consumption may have increased the secretion rate of salivary HBD2, which, in turn, may have influenced the *Oribacterium*, an oral commensal bacterium, in the tongue-coating microbiota (Figure 4). Notably, there was no difference in the proportion of *Oribacterium* in the tongue-coating microbiota between the yogurt and non-yogurt groups (Table 3). The genus *Oribacterium* consists of obligate anaerobic bacteria that constitute the normal microbiota of the tongue coating in healthy individuals [59]. Salivary HBD2 exhibits functions beyond antimicrobial activity, including immune modulation and inflammation control [60]. Notably, these elevated levels associated with LbR1 yogurt consumption might potentially contribute to maintaining the relative abundance of *Oribacterium* within the tongue coating.

Bayesian network analysis revealed that LbR1 yogurt consumption may trigger sequential interactions among salivary AMPs (Figure 4). Specifically, the IgA secretion rate affected that of HBD2 (Figure 4). Notably, IgA is the most abundant antibody on mucosal surfaces, preventing the adhesion of microorganisms to the mucosal epithelium and playing a crucial role in infection defense [61]. Salivary IgA and HBD2 are both secreted in response to common stimuli, such as microbial challenge and inflammatory cytokines such as IL-1β and TNF-α [60,62]. Therefore, it is likely that the IgA flow rate influenced the HBD2 flow rate. Additionally, the HBD3 flow rate influenced the lactoferrin flow rate, with a positive correlation observed between the two (Figure 4). Following yogurt consumption, epithelial cells in the oral mucosa secrete HBD3, which serves as a signal to activate immune cells such as neutrophils [63]. Activated neutrophils release large amounts of stored lactoferrin [64], potentially increasing the rate of lactoferrin secretion into saliva. The salivary lactoferrin flow rate, in turn, affected the salivary IgA flow rate, with a positive correlation observed between the two (Figure 4). Notably, salivary lactoferrin and IgA have been reported to be regulated by a common mechanism [65]. Therefore, here, salivary lactoferrin and IgA may have been secreted cooperatively as components of the oral mucosal defense system. Although most salivary antibacterial proteins are ineffective at low concentrations, they synergize to form an efficient defense network within the oral cavity [66]. Therefore, LbR1 yogurt consumption might stimulate oral immune system, potentially contributing to the defense against oral infections.

Bayesian network analysis revealed that both the salivary lactoferrin flow rate and age influenced the salivary flow rate (Figure 4). Specifically, salivary lactoferrin flow rate and age demonstrated a positive and negative association with the salivary flow rate, respectively (Figure 4). In an Alzheimer’s mouse model, salivary lactoferrin levels were reduced, and concentrations of acetylcholine, a parasympathetic neurotransmitter, in the submandibular gland were also decreased [67]. Moreover, salivary secretion is promoted by activation of the parasympathetic nervous system [68]. A previous study by Romão da Silva et al. reported that continuous intake of lactic acid bacteria for 8 weeks results in the activation of the parasympathetic nervous system [69]. Thus, continued LbR1 yogurt consumption and parasympathetic nervous system activation due to elevated lactoferrin levels positively influenced the salivary flow rate. Notably, the salivary flow rate decreases with age [70]. Additionally, the Bayesian network results indicated that increasing age negatively affected the salivary flow rate (Figure 4), which in turn increases respiratory infection risk [71]. Here, the yogurt group was 15 years older than the non-yogurt group; nonetheless, there was no significant difference in the median salivary flow rate (Table 1). Older adult individuals with reduced salivary flow rates may benefit from consuming LbR1 yogurt, as it increases lactoferrin levels in the saliva and activates the parasympathetic nervous system, potentially helping to prevent xerostomia and respiratory infections. While we observed an association between lactoferrin and salivary flow rate, we did not directly measure nerve activity. Therefore, the involvement of the parasympathetic nervous system to explain the maintained salivary flow rate in the older yogurt group remains a hypothesis based on previous reports.

Bayesian network analysis revealed that LbR1 yogurt consumption may have influenced URTIs, with the yogurt group exhibiting a lower number of URTIs compared to the non-yogurt group (Table 1). Several reports have indicated that lactic acid bacterial intake reduces URTI frequency [72,73]. Specifically, a previous study by Makino et al. reported that exopolysaccharide (EPS) produced by LbR1 activates NK cells [16]. Although we did not assess NK cell function, this previously established mechanism might underlie the reduced URTI frequency observed in our yogurt group. Furthermore, viral infections, such as URTIs, increase in both incidence and severity with advancing age in adults [74]. In this study, although the median age of participants in the yogurt group was 15 years greater than that of participants in the non-yogurt group, the number of URTIs was lower (Table 1). These results suggest that consumption of probiotics, such as LbR1 yogurt, might potentially contribute to reducing the frequency of URTIs in older adults.

Our Bayesian network analysis revealed that LbR1 yogurt consumption may have influenced the abundance of *Haemophilus* in the tongue coating (Figure 4). Specifically, the proportion of *Haemophilus* in the yogurt group was lower than that in the non-yogurt group, albeit this difference was not statistically significant (Table 2). *Haemophilus* are commensal oral bacteria that prefer neutral to alkaline conditions and can act as opportunistic pathogens [75,76]. Consuming LbR1 yogurt has been reported to activate innate immunity, such as NK cells [16]. Thus, LbR1 yogurt consumption might potentially be associated with a lower proportion of *Haemophilus* in the tongue coating, possibly mediated by the activation of the oral innate immune system; however, as no significant differences were observed, the impact on this specific genus appears to be limited.

In all, our findings suggest a potential mechanistic pathway linking LbR1 yogurt consumption to reduced URTI frequency. The continuous intake of LbR1 may stimulate oral epithelial cells and leukocytes, leading to the upregulated secretion of AMPs such as HBD2, HBD3, and lactoferrin. This enhanced innate immune response, possibly coupled with environmental changes (e.g., pH modulation), appears to create conditions that disfavor specific pathobionts like *F. nucleatum* ssp. *animalis*, while allowing commensal bacteria such as *Lachnoanaerobaculum* to thrive. Collectively, this dual action—bolstering the mucosal immune barrier and optimizing the tongue microbiota profile—may contribute to the observed lower frequency of URTIs. However, the Bayesian network analysis did not indicate that the salivary AMPs elevated by LbR1 yogurt intake directly influenced oral bacteria or URTIs; therefore, further research is required to verify direct causal relationships. Furthermore, particularly considering that this study is an observational study with a small sample size (*n* = 53), the relationships identified through the Bayesian network analysis should be viewed as hypothesis-generating.

This study has several limitations inherent to its observational pilot design. First, the sample size was small (*n* = 53) and unbalanced between the yogurt group (*n* = 40) and the non-yogurt group (*n* = 13). Furthermore, there was a significant age difference between the groups, with the yogurt group being older. To mitigate the risk of false positives associated with the small and unbalanced sample size, we employed the non-parametric Mann–Whitney U test, which has been reported to avoid Type I error inflation under such conditions [77]. However, due to the lack of a formal power calculation prior to recruitment, the statistical power may be limited. Consequently, the statistical analysis of the tongue-coating microbiota was conducted in an exploratory manner. Additionally, although we incorporated age as a factor in the Bayesian network analysis to address confounding, the relationships identified should be viewed as hypothesis-generating rather than confirmatory, particularly given the cross-sectional nature of the data. Future studies involving a larger number of participants and employing a multicenter, double-blind, parallel-group, randomized controlled trial that considers both participant number and age matching are warranted.

Second, methodological limitations regarding microbiome analysis must be acknowledged. We sampled only the tongue coating based on the hypothesis that it interacts directly with saliva; however, the oral microbiome is site-specific, and the tongue coating profile may not fully reflect the microbiota of other niches such as dental plaque or saliva itself [78]. Analytically, we used 16S rRNA gene sequencing targeting the V1–V2 region. While effective for genus-level identification, shotgun metagenomic sequencing would provide superior resolution for species-level and functional analysis [79,80,81]. Moreover, caution is warranted when interpreting the biological significance of taxa with low relative abundance identified in this study (e.g., *Lachnoanaerobaculum*, Ruminococcaceae G2, and *F. nucleatum* ssp. *animalis*), as rare taxa can exhibit instability in amplicon sequencing data. Furthermore, this study did not assess the prevalence of periodontal or cariogenic bacteria other than *Fusobacterium* species.

Third, we examined the effects of consuming LbR1 yogurt as a whole, and thus could not isolate the specific effects of the LbR1 strain from the yogurt matrix or other ingredients. Regarding the clinical outcome, the frequency of URTIs was defined as the number of doctor-diagnosed episodes within the past year. Although the nursing home director verified these self-reported questionnaire responses to ensure accuracy, the retrospective nature of the assessment introduces the potential for recall bias. Finally, the participants were nursing home care workers, a specific population potentially subject to higher exposure to infectious agents and occupational stress compared to the general public. Therefore, the generalizability of these findings to other populations remains to be verified.

## 5. Conclusions

The continuous consumption of LbR1 yogurt is associated with elevated salivary secretion rates of the AMPs HBD2 and HBD3 and decreased URTI frequency. In the LbR1 yogurt intake group, the proportion of *F. nucleatum* ssp. *animalis* associated with colorectal cancer in the tongue-coating microbiota was lower than that in the non-yogurt group. Thus, LbR1 yogurt intake is associated with modulations in oral innate immunity markers and specific tongue microbiota changes, suggesting a potential link to the observed lower frequency of URTIs. However, as this study is an observational pilot study, the results should be interpreted with caution.

## Figures and Tables

**Table 1:** General data of participants.

Parameter	Non-Yogurt Group (*n* = 13)	Yogurt Group (*n* = 40)	*p*-Value
Sex (*n*): Female/male	6/7	33/7	
Age (year)	39 (31–48)	54 (39–66)	0.03 *
BMI (%)	19.1 (17.4–24.7)	22.2 (19.9–25.3)	0.1
Number of teeth	28.0 (26.0–28.0)	28.0 (26.3–28.0)	0.8
Daily sleep duration (h)	6.5 (6.0–7.0)	6.0 (6.0–7.0)	0.3
Total number of COVID-19 infections (times)	1 (0–1)	1 (0–1)	0.4
Number of URTI (times/year)	0 (0–1) Average: 0.215	0 (0–0) Average: 0.025	0.003 *
Current subjective health status (−7 to 7)	1.1 (0–5.4)	0.6 (0–3.1)	0.3
Salivary flow rate (mL/min)	0.669 (0.460–0.764)	0.619 (0.348–0.745)	0.6

BMI, body mass index; COVID-19, coronavirus disease 2019; URTI, upper respiratory tract infection; current subjective health status, values were evaluated in increments of 0.1 from the minimum value of −7 to the maximum value of +7. All variables, except sex, are expressed as the median (first quartile–third quartile). Statistical analysis was performed using the Mann–Whitney U test (* *p* < 0.05). The number of URTIs is also presented as the average value.

**Table 2:** Abundance of the top 25 bacterial genera in the tongue-coating microbiota (%).

Genus	Non-Yogurt Group (*n* = 13)	Yogurt Group (*n* = 40)	*p*-Value
*Streptococcus*	33.3 (20.4–42.6)	26.6 (18.7–32.9)	0.3
*Neisseria*	8.50 (2.23–26.5)	12.5 (2.10–22.3)	1.0
*Veillonella*	6.44 (5.24–8.78)	8.19 (5.04–10.2)	0.3
*Prevotella*	5.50 (2.89–7.69)	7.84 (5.11–17.5)	0.1
*Schaalia*	7.58 (3.60–10.8)	6.91 (4.56–9.17)	1.0
*Rothia*	8.02 (5.43–10.7)	6.11 (3.28–9.57)	0.3
*Granulicatella*	5.34 (2.15–6.57)	4.46 (2.85–5.36)	0.6
*Haemophilus*	4.15 (1.94–6.43)	2.71 (1.06–5.78)	0.1
*Fusobacterium*	1.95 (0.58–5.39)	2.11 (0.94–3.61)	0.6
*Porphyromonas*	1.10 (0.46–5.34)	1.94 (0.57–3.56)	1.0
*Alloprevotella*	0.62 (0.23–0.98)	1.41 (0.34–2.70)	0.1
*Gemella*	1.54 (0.99–2.34)	1.40 (0.90–2.53)	0.9
*Actinomyces*	1.17 (0.46–1.50)	1.11 (0.60–2.17)	0.6
*Leptotrichia*	0.54 (0.42–0.91)	0.88 (0.59–1.55)	0.1
*Lachnoanaerobaculum*	0.19 (0.12–0.26)	0.43 (0.21–0.80)	0.004 *
*Stomatobaculum*	0.13 (0.075–0.72)	0.40 (0.16–0.92)	0.1
*Oribacterium*	0.37 (0.22–0.48)	0.37 (0.26–0.57)	0.3
*Atopobium*	0.16 (0.040–0.28)	0.33 (0.11–0.87)	0.1
*Campylobacter*	0.25 (0.15–0.38)	0.30 (0.16–0.38)	0.5
*Solobacterium*	0.20 (0.10–0.34)	0.27 (0.11–0.72)	0.3
*Capnocytophaga*	0.13 (0.035–0.32)	0.18 (0.023–0.46)	0.7
*Catonella*	0.07 (0.035–0.09)	0.08 (0.02–0.13)	0.6
Ruminococcaceae_G2	0.01 (0.005–0.03)	0.045 (0.03–0.12)	0.004 *
*Tannerella*	0.03 (0.02–0.09)	0.035 (0.01–0.078)	0.4
*Treponema*	0.08 (0.01–0.23)	0.015 (0–0.075)	0.06

All variables are expressed as median (first quartile–third quartile), and statistical analysis was performed using the Mann–Whitney U test (* *p* < 0.05).

**Table 3:** Abundance of major *Fusobacterium* species in the tongue-coating microbiota (%).

Species	Non-Yogurt Group (*n* = 13)	Yogurt Group (*n* = 40)	*p*-Value
*Fusobacterium hwasookii*	0 (0–0)	0 (0–0.02)	0.2
*Fusobacterium nucleatum* ssp. *animalis*	0.04 (0.02–0.09)	0.02 (0–0.05)	0.04 *
*Fusobacterium nucleatum* ssp. *nucleatum*	0 (0–0)	0 (0–0)	0.7
*Fusobacterium nucleatum* ssp. *polymorphum*	0 (0–0.04)	0 (0–0.04)	1.0
*Fusobacterium nucleatum* ssp. *vincentii*	0.01 (0.005–0.2)	0.02 (0–0.08)	0.5
*Fusobacterium periodonticum*	1.8 (0.31–4.4)	1.5 (0.76–3.2)	0.6
*Fusobacterium* sp. HMT 203	0 (0–0.01)	0 (0–0)	0.3
*Fusobacterium* sp. HMT 204	0 (0–0)	0 (0–0)	0.8
*Fusobacterium* sp. HMT 248	0 (0–0)	0 (0–0)	0.6

All variables are expressed as median (first quartile–third quartile), and statistical analysis was performed using the Mann–Whitney U test (* *p* < 0.05).

## Data Availability

All data obtained in this study are described in the manuscript.

## Abbreviations

The following abbreviations are used in this manuscript:

AMP	Antimicrobial Protein
BMI	Body Mass Index
COVID-19	Coronavirus Disease 2019
ELISA	Enzyme-linked immunosorbent assay
EPS	Exopolysaccharide
FDR	False Discovery Rate
HBD	Human β-defensin
IgA	Immunoglobulin A
IL-1β	Interleukin-1 beta
LbR1	*Lactobacillus delbrueckii* ssp. *bulgaricus* OLL1073R-1
NK	Natural Killer
PCoA	Performing principal Coordinate Analysis
PERMANOVA	Permutational Multivariate Analysis of Variance
TNF-α	Tumor Necrosis Factor Alpha
URTI	Upper Respiratory Tract Infection
